# Alternative Splicing (AS) Provides an Alternative Mechanism for Regulating GLIS3 Expression and Activity

**DOI:** 10.3390/cells14231912

**Published:** 2025-12-02

**Authors:** David W. Scoville, Sara A. Grimm, Jason G. Williams, Anton M. Jetten

**Affiliations:** 1 Cell Biology Group, Immunity, Inflammation and Disease Laboratory, National Institute of Environmental Health Sciences, National Institutes of Health, Durham, NC 27709, USA; 2 Integrative Bioinformatics Support Group, National Institute of Environmental Health Sciences, National Institutes of Health, Durham, NC 27709, USA; 3 Mass Spectrometry Research and Support Group, National Institute of Environmental Health Sciences, National Institutes of Health, Durham, NC 27709, USA

**Keywords:** GLIS3, isoforms, transactivation, RIME

## Abstract

**Highlights:**

**What are the main findings?**

- We identified a new isoform of the mouse *Glis3* gene that is the predominant isoform in most tissues.
- We identified several potential mechanisms for the post-translational control of GLIS3.

**What is the implication of the main finding?**

- We identify different isoforms, post-translational modifications, and protein interactions for GLIS3 which offer mechanisms for regulating its activity and physiological processes, as well as its role in disease.

**Abstract:**

The Krüppel-like transcription factor GLIS3 plays an important regulatory role in the development of various tissues, both in mice and humans. Loss-of-function mutations in *GLIS3* are implicated in several pathologies, including polycystic kidney disease, diabetes, and hypothyroidism. Previous studies have reported that the mouse *Glis3* gene generates a 7524 bp mRNA encoding a 935 amino acid (aa) protein, with a homologous human protein of 930 aa. Here, we identify a shorter mouse mRNA lacking the third exon, producing a shorter 659 aa GLIS3 protein. This shorter transcript is expressed at a higher level than the longer transcript in all mouse tissues tested and produces a protein that is more stable and exhibits a greater transactivation potential. This suggests that the 276 aa N-terminus in the longer mouse GLIS3 protein encompasses important regulatory domain(s). Mass spectrometry identified several phosphorylation sites that may contribute to the post-translational regulation of GLIS3 activity and function and several known members of co-activator and co-repressor complexes, consistent with the concept that GLIS3 can act both as a transcriptional repressor and activator. These data offer important insights into how GLIS3 activity is regulated and offer potential mechanisms for its control during tissue development and disease.

## 1. Introduction

*Glis3* was first identified in 2003 in mice, encoding a protein with five C_2_H_2_-type zinc fingers with a high homology to those of GLI and ZIC proteins [1]. A subsequent analysis showed that the mouse *Glis3* genome sequence produces a 935 aa protein [2]. This GLIS3 protein (and its human homolog containing 930 aa) localizes to the nucleus, where it functions as an activator or repressor of gene transcription [2]. Subsequent studies have implicated GLIS3 in the regulation of many biological functions, including thyroid hormone biosynthesis, pancreatic β cell generation and insulin gene production, and the maintenance of renal functions, and in various pathologies, including diabetes, polycystic kidney disease, hypothyroidism, and several malignancies [3,4,5,6,7,8,9]. Loss-of-function mutations in *GLIS3* are causally linked to neonatal diabetes and hypothyroidism (NDH) in humans [10,11,12,13,14,15,16], matching the phenotype observed in mice with deletions of *Glis3* [3,4,5,6,8]. Additionally, SNPs in presumed regulatory regions within the first and second intron of *GLIS3* have been associated with an increased risk of both Type 1 and Type 2 diabetes (reviewed in [9]). Chromosomal rearrangements of the *GLIS3* locus have also been implicated in various cancers [7]. Taken together, these studies indicate that GLIS3 plays a critical role in the regulation of an increasing number of biological functions in various tissues and in the development of several diseases.

Although great insights have been obtained into the mechanism by which GLIS3 regulates various biological functions and gene transcription [7,8,9], little is known about the mechanisms that regulate GLIS3 expression and activity. In addition to transcriptional, translational, and post-translational regulation, the alternative splicing (AS) of many genes has been shown to provide alternative mechanisms for regulating gene expression and cellular processes [17]. AS allows the generation of different unique transcripts from a single gene and the generation of different protein isoforms that might differ in stability, localization, and function. In this study, we identify an AS variant of *Glis3* via an RNA-seq analysis of mouse testis, kidney, thyroid, and pancreatic islets. This shorter splice variant lacks exon 3, causing a translation from the original methionine, resulting in a frameshift and premature stop codon in exon 4. This alternative transcript encodes a smaller protein of 659 amino acids (aa) that lacks the first 276 aa of the long GLIS3 protein. Although many tissues express both transcripts, the “short” splice variant is expressed at a higher level than the “long” variant. We provide evidence showing that the short variant of the GLIS3 protein is more stable and exhibits a greater transcriptional activity, suggesting that the 276 aa N-terminal region contains regulatory region(s) (e.g., degron) or post-translational modifications that affect GLIS3 activity. Mass spectrometric analysis identified several post-translational modifications in the GLIS3 N-terminal region that might impact GLIS3 activity and function. In addition, this analysis identified several co-activators and co-repressors as part of GLIS3 transcriptional complexes. The latter is in line with observations that GLIS3 can both act as a repressor and activator of gene transcription [1,2]. We propose that the different isoforms, the post-translational modifications, and protein interactions together define GLIS3 activity and its regulation of physiological processes, as well as its role in disease.

## 2. Materials and Methods

### 2.1. Analysis of Intron–Exon Junctions in RNA-Seq Data

RNA-seq data were analyzed from existing in-house datasets (GEO accessions GSE122120, GSE207775, and GSE240532) limited to wild-type animals only. Raw sequenced reads were mapped to the mm10 reference assembly via STAR v2.7.11b [18] with parameters “--twopassMode Basic --outFilterType BySJout --alignSJoverhangMin 8 --outFilterMultimapNmax 20 --outFilterMismatchNmax 999 --outFilterMismatchNoverReadLmax 0.04 --alignSJDBoverhangMin 1 --alignIntronMin 20 --alignIntronMax 1000000 --alignMatesGapMax 1000000”. Counts of mapped fragments crossing annotated exon junctions (exon2/exon4 in support of the short *Glis3* isoform or the average of exon2/exon3 and exon3/exon4 in support of the long *Glis3* isoform) were collected from the STAR splice junction outputs, then scaled to 50 million uniquely mapped fragments per sample. Publicly available RNA-seq data from pigs (kidney: GSE162148; testis: GSE162148, GSE73763, GSE76665, GSE233410), rhesus monkeys (kidney: GSE162142, GSE114191; testis: GSE97786, GSE69241, GSE34426), dogs (kidney: GSE162142, GSE203106, GSE106077; testis: GSE106077, PRJNA901164), and rabbits (kidney: GSE162142, GSE106077, GSE114191; testis: GSE106077, PRJNA1154456) were processed as described above for the mouse data, with alignment against the susScr11, rheMac10, canFam4, and oryCun2 genomes, respectively.

### 2.2. qRT-PCR Analysis

Primers for qRT-PCR analysis were designed targeting mouse *Glis3* exons 2, 3, and 4, and for human *GLIS3* targeting exons 2, 3, and an alternative exon 1 for the short isoform (Supplementary Table S1). For experiments using mouse tissue, WT mice on a C57BL/6 background routinely fed an NIH-31 diet (Harlan Labs, Indianapolis, IN, USA) were used. Db/db mice were obtained from Jackson Laboratory, #000697 (Bar Harbor, ME, USA). Tissue collection followed the guidelines outlined in the NIH Guide for the Care and Use of Laboratory Animals, and the protocols were approved by the Institutional Animal Care and Use Committee at the National Institute of Environmental Health Sciences (NIEHS) (Approval #P05-46), RNA was isolated using a combination of Trizol reagent (Life Technologies, Carlsbad, CA, USA) and column DNAse digestion using RNeasy Mini kits (Qiagen, Germantown, MD, USA). Pancreatic islet RNA was isolated as previously described [19]. Pooled human RNA from different tissues was purchased commercially (#636643, Clontech, Mountain View, CA, USA). cDNA was synthesized using a High Capacity DNA Reverse Transcription Kit (Applied Biosystems, Foster City, CA, USA). qRT-PCR was carried out in triplicate in a 7300 Real Time PCR System (Applied Biosystems, Foster City, CA, USA). All results were normalized to *18S* expression and are shown relative to the control using the 2^ΔΔct^ method.

### 2.3. Expression Plasmids

The expression plasmids p3XFlag-CMV-10-mGLIS3L (long isoform—mouse), p3XFlag-CMV-10-mGLIS3S (short isoform—mouse), p3XFlag-CMV-10-hGLIS3L (long isoform—human), and truncations GLIS3 ΔN70, ΔN140, and ΔN210 were produced through PCR amplification (Supplementary Table S1) and inserted into p3XFlag-CMV-10 (Sigma, St. Louis, MO, USA). Flag-hGLIS3S (short isoform—human) cloned into the p3XFlag-CMV-10 expression plasmid and the luciferase reporter (GLISBS-Luc) under the control of the GLIS binding site (GLISBS) were previously described [2]. Constructs were verified via DNA sequence analysis.

### 2.4. Luciferase Assays

Luciferase assays were performed as previously described [4]. Briefly, HEK293T cells were plated at 1 × 10^5^ in 24-well plates and transfected the following day with 50 ng GLISBS-Luc, 50 ng CMV-β-galactosidase, 100 ng of empty p3XFlag-CMV-10, or the indicated GLIS3 plasmid using Lipofectamine 2000 (Life Technologies, Carlsbad, CA, USA) following the manufacturer’s protocol. The following day, cells were lysed in Passive Lysis buffer (Promega, Madison, WI, USA), and reporter activity was measured with a luciferase assay kit (Promega, Madison, WI, USA). β-galactosidase activity was measured using a luminometric β-galactosidase detection kit (Clontech, Mountain View, CA, USA), and luciferase activity was normalized to β-galactosidase activity. Each condition was tested in triplicate, and each experiment was repeated at least three times. Error bars indicate the standard error of the mean.

### 2.5. Stability Assays

HEK293T cells were transfected with either p3XFlag-CMV-10-mGLIS3S or p3XFlag-CMV-10-mGLIS3L in a 6-well plate using Lipofectamine 2000 (Life Technologies) following manufacturer’s protocol. After 24 h, cells were treated with cycloheximide (100 μg/mL) for the indicated times, then nuclear extract was collected as previously described [20]. The protein concentrations of the resulting extract were measured, and equal amounts of protein were used for Western blot analysis. Blots were probed with the Flag antibody (mouse, #F1804, at 1:2000, Sigma, St. Louis, MO, USA) and β-actin (mouse #4970, at 1:4000, Cell Signaling, Danvers, MA, USA). Images of blots were captured on an iBright system (# A32752, Life Technologies, Carlsbad, CA, USA), and densitometry was measured via FIJI [21]. Flag signal was normalized to the β-actin signal, and error bars indicate the standard error of the mean.

### 2.6. Immunoprecipitation and Mass Spectrometry

βTC-6 cells were maintained in DMEM high glucose (#11965, Life Technologies, Carlsbad, CA, USA) supplemented with 10% FBS and penicillin/streptomycin. Briefly, a plasmid construct containing the mouse *Glis3* short or long isoforms was tagged at the N-terminus with Flag and the C-terminus with HA. These *Glis3* constructs were then cloned into the pInducer20 plasmid, which contains a doxycycline-inducible promoter, and then used to produce lentivirus (pInd20-GLIS3). βTC-6 cells were infected with pInd20-GLIS3 lentivirus at a multiplicity of infection (MOI) of 1. Infected cells were subsequently selected using G418 (Geneticin, Life Technologies # 10131035) at 500 μg/mL for ~1 week. G418 selected cells were expanded and treated for 24 h with doxycycline (100 ng/mL) in a tetracycline-negative medium. Doxycycline-treated non-infected βTC-6 cells were used as a control. Subsequently, βTC-6 cells (45–60 million) were collected, nuclear protein was extracted, and GLIS3 protein complexes were isolated through immunoprecipitation with either Flag- or HA-bound magnetic beads (# A36797 or 88837, respectively, Life Technologies, Carlsbad, CA, USA) as described previously [22]. For the Rapid immunoprecipitation mass spectrometry of endogenous proteins (RIME) [23], cells were crosslinked with 1% formaldehyde for 10 min and sonicated using a Focused Ultrasonicator (#E220, Covaris, Woburn, MA, USA), and cell extracts were processed for immunoprecipitation as described above. The resulting immunoprecipitates were directly digested from beads using the Thermo Fisher Scientific (Rockford, IL, USA) EasyPep Mini MS sample preparation kit essentially following the manufacturer’s instructions, except performing protein digestion and transfer to a new tube prior to the reduction and alkylation of cysteines. Mass spectrometry measurements and database searching were performed as previously described [24].

### 2.7. Statistical Analysis

Where indicated, statistics were calculated using a two-tailed Student *t*-test. * indicates a *p*-value < 0.05, ** *p* < 0.01, and *** *p* < 0.001.

## 3. Results

### 3.1. Identification of GLIS3 Isoforms in Mice and Humans

**Figure 1:**
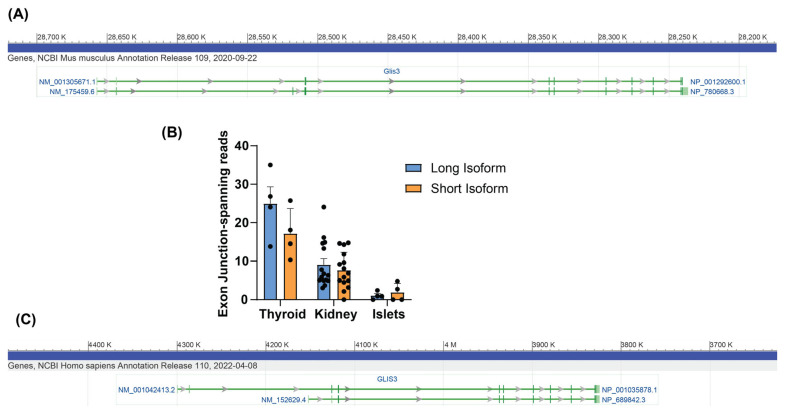
Two isoforms of *Glis3* exist in mice. (**A**) NCBI annotates two isoforms of *Glis3*, and (**B**) RNA-seq analysis of *Glis3* expression shows that both isoforms are expressed. Graph represents # of exon-spanning reads from exon 2–3 and 3–4 (long isoform), or exon 2–4 (short isoform). Long isoform reads were divided by 2. Reads are taken from three independent samples for each organism; error bars indicate SEM. (**C**) NCBI annotates two isoforms for human *GLIS3*, with the shorter isoform starting from an alternative exon 1 and splicing to exon 3.

The analysis of the RefSeq database suggested that the mouse *Glis3* gene may undergo AS to produce two transcripts encoding two different protein isoforms (Figure 1A). The longest splice variant (NM_175459.6) produces the largest protein, which is generally considered the canonical GLIS3 mouse protein, as the homologous *GLIS3* gene in humans produces a protein of a similar size. The second isoform (NM_001305671.1) lacks exon 3, and translation begins at a methionine in the 4th exon, producing a protein that is 276 aa shorter than the longest isoform. The analysis of exon-spanning reads from mouse RNA-seq data reveals that both RNA isoforms are transcribed in vivo in mice (Figure 1B). Interestingly, while the long isoform appears conserved in humans, no homolog to the mouse short isoform has been identified, likely due to a lack of the corresponding methionine in the human sequence. However, a shorter isoform of the human protein has been annotated (Figure 1C), which starts from an alternative exon 1 prior to exon 3. This produces a protein which is 155 aa shorter. The RNA-seq analysis of publicly available human pancreatic islet data failed to identify any exon-spanning reads that could be attributable to this shorter isoform (ArrayExpress Accession #E-MTAB-5060). Thus, the longer transcript and the larger isoform of GLIS3 may be the only transcript and protein produced in the normal human pancreas.

### 3.2. The Mouse Glis3 Short Isoform Is Expressed at Higher Levels than the Long Isoform

**Figure 2:**
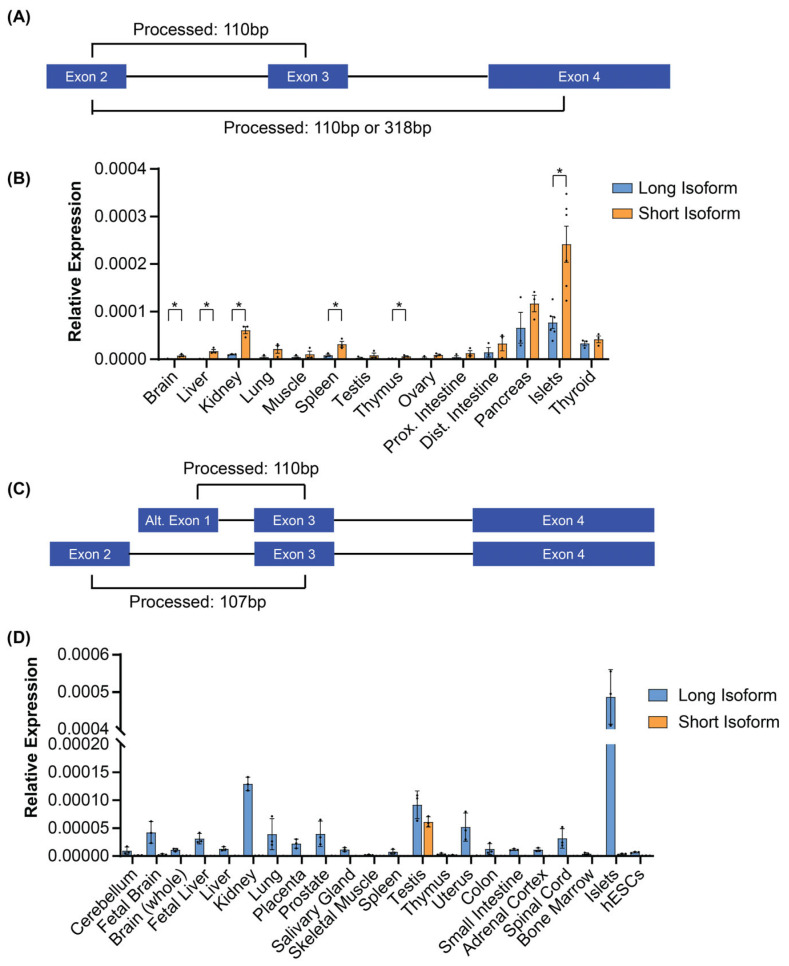
Mouse *Glis3* and human *GLIS3* short and long isoform expression in a variety of tissues. (**A**) Design of qPCR primers to detect mouse *Glis3* long and short isoform. (**B**) qPCR expression results for *Glis3* isoforms in a variety of tissues. Error bars indicate SEM, N = 3. * indicates a *p*-value < 0.05. (**C**) Design of qPCR primers to detect human *GLIS3* long and short isoform. (**D**) qPCR expression results for *GLIS3* isoforms in human tissues. Error bars indicate SEM from technical replicates of pooled tissue RNA.

To examine the expression of the long and short *Glis3* isoforms identified in Figure 1, qPCR primers were designed to distinguish the two isoforms (Figure 2A). As it is possible for the primer ending in exon 4 to also amplify a larger product including exon 3, we limited the extension time of the PCR and therefore did not detect this larger product (Supplementary Figure S1). Our analysis showed that both *Glis3* short and long transcripts were expressed across a variety of mouse tissues (Figure 2B). This analysis further indicated that, while both long and short variants are expressed, the shorter variant appears to be expressed at higher levels than the longer variant in some tissues (brain, liver, kidney, spleen, thymus, and pancreatic islets). Next, we analyzed the expression of the human short and long *GLIS3* transcript in several human tissues using respective qPCR primers (Figure 2C). Interestingly, except for the testis, the long isoform was the only isoform detected in all tissues analyzed, including the pancreas (Figure 2D). The latter is consistent with our earlier RNA-seq analysis of human pancreatic islets, in which we were unable to identify alternative exon 1-to-exon 3 spanning reads. Thus, in contrast to mice, human tissues generally express one isoform, except for in the testis.

Dysregulated splicing events have been implicated in various pathologies, including cancer, neurological disorders, and the pathophysiology of pancreatic cells [25,26,27]. As GLIS3 plays a critical role in pancreatic beta cells and diabetes [4,5,10,11,19], we examined this possibility in pancreatic islets from wild-type mice and mice with a genetic disruption of the leptin receptor (*db*/*db*), which leads to a diabetic phenotype. Although these pancreatic islets showed a trend towards reduction in the total levels of *Glis3*, the reduction was similar between the two isoforms (Supplementary Figure S2).

### 3.3. The Short Mouse Isoform of GLIS3 Exhibits Greater Activity and Stability

**Figure 3:**
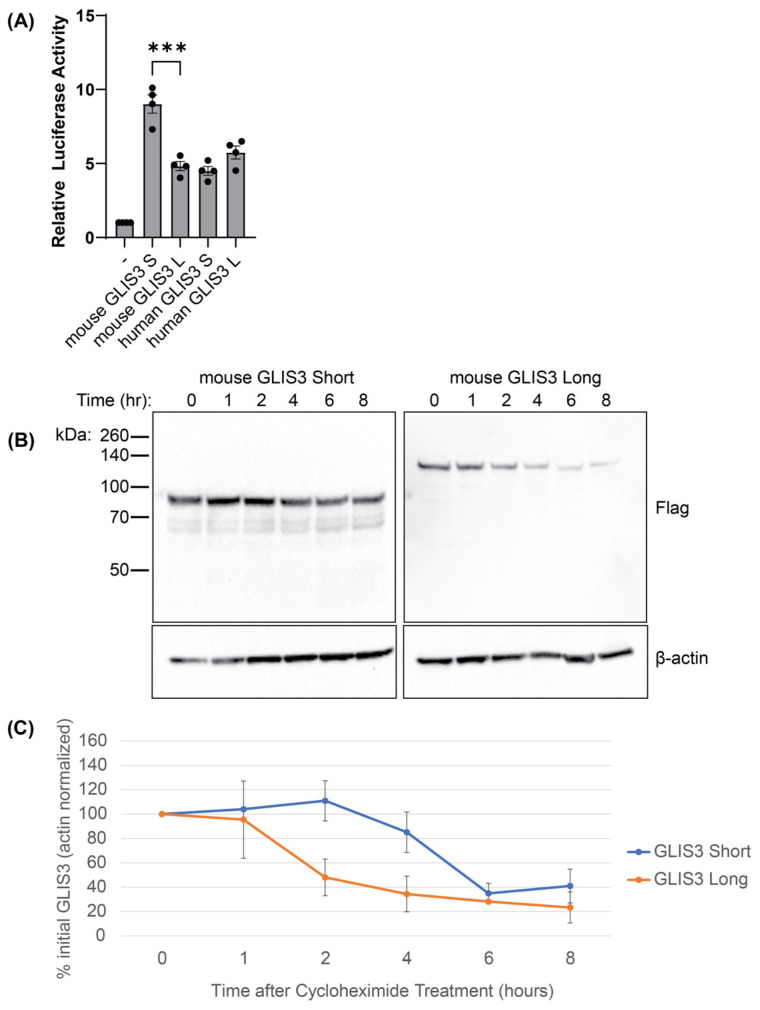
The mouse GLIS3 short isoform has more transactivation potential and stability than the long isoform. (**A**) Luciferase assays examining transactivation potential of mouse GLIS3 long and short isoforms and human long and short isoforms. Error bars indicate standard deviation. *** indicates a *p*-value < 0.001. (**B**) Representative Western blots of Flag-tagged GLIS3 short and long isoforms. Antibodies for Flag and β-actin were used, as indicated. Molecular weights are shown to the left for the Flag blot. (**C**) Quantification of GLIS3 levels (as measured by Flag staining) normalized to β-actin levels. Error bars indicate SEM, N = 3.

Isoforms generated by alternative splicing have been implicated in the regulation of distinct physiological and developmental processes [17]. This raised the question of whether the two GLIS3 isoforms have different activities and physiological functions. The specific expression of the human short GLIS3 isoform in the testis suggests a possible tissue-specific role. To determine whether the GLIS3 isoforms exhibited any functional difference, their transcriptional activities were compared in a reporter assay in which the luciferase reporter activity was under the control of GLISBS. This analysis revealed that the mouse short isoform had roughly twice the transactivation capability of the mouse long isoform (Figure 3A). The transactivation activity of the human short and long isoform was very similar and comparable to that of the mouse long isoform. One possible explanation for the difference in activity between the short and long mouse isoform would be a difference in protein stability. To test this, HEK293T cells expressing either the long or short isoform of mouse GLIS3 were treated with cycloheximide, an inhibitor of RNA translation, which allows for the measurement of protein degradation. This analysis showed that the GLIS3 short isoform was more stable than the long isoform (Figure 3B,C), indicating that the first 276 aa of the mouse GLIS3 long isoform plays an important role in protein stability, although we cannot rule out additional functions in transactivation.

### 3.4. The N-Terminal Region Between 140 and 210 Is Responsible for the Difference in Activity Between GLIS3 Long and Short Isoforms

**Figure 4:**
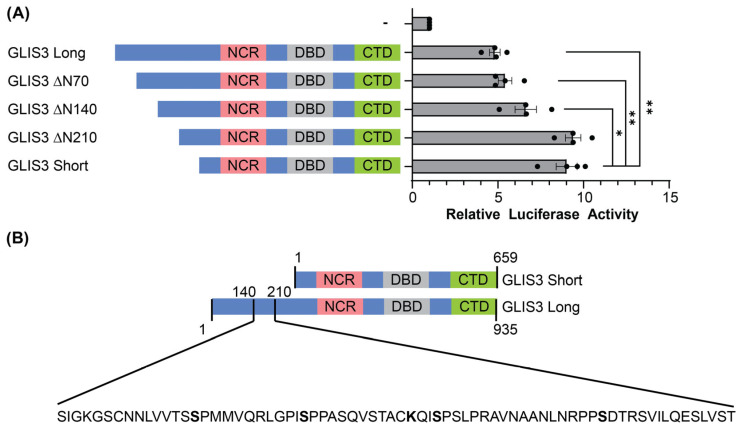
Difference between mouse GLIS3 long and short isoforms resides in aa 140-210. (**A**) Luciferase assays with truncations of the mouse GLIS3 long isoform and the GLIS binding site (GLISBS) luciferase reporter. Error bars indicate standard deviation. * indicates a *p*-value < 0.05; ** < 0.01. (**B**) AA 140-210 in the GLIS3 long isoform. NCR = N-terminal conserved region, DBD = DNA binding domain, CTD = C-terminal domain.

To narrow down the specific aa responsible for the differences in transactivation, truncation mutants were made of the GLIS3 long isoform. Truncations lacking the first 70 or 140 aa of the long isoform had little effect on the transactivation activity (Figure 4A). However, a truncation of the first 210 aa did increase the activity to a level similar to that of the short isoform. This indicates that a potential repressive domain of GLIS3 resides within the region between 140 and 210 aa (Figure 4B).

### 3.5. GLIS3 Is a Heavily Phosphorylated Protein and Interacts with Several Proteins

**Figure 5:**
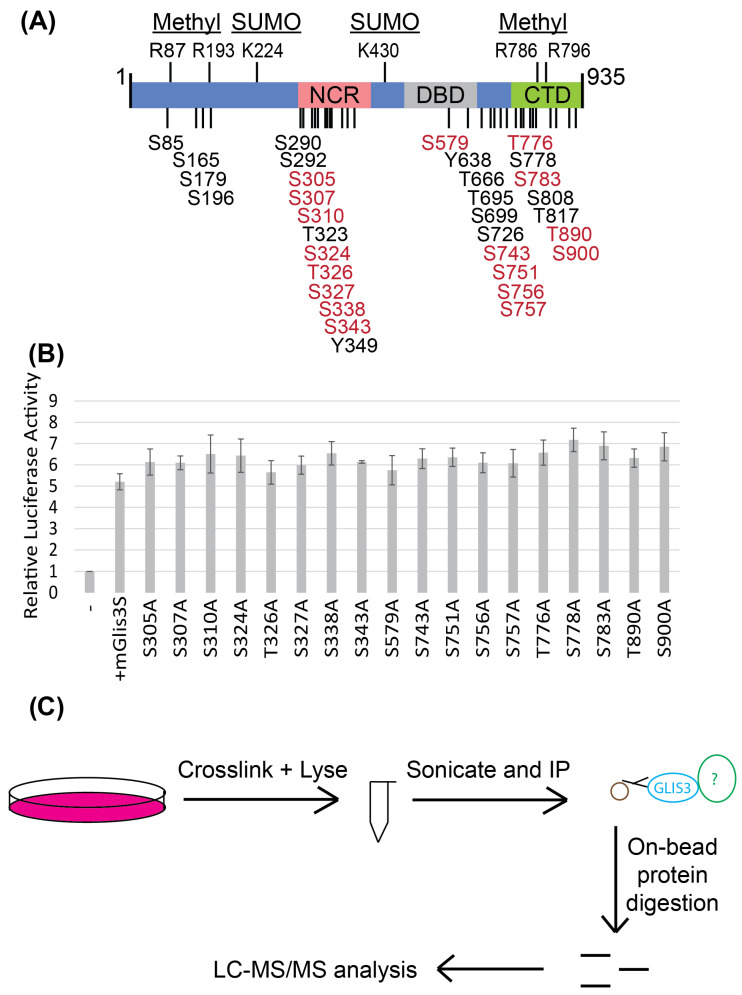
Identification of additional GLIS3 phosphorylation sites. (**A**) Representation of GLIS3 aa sequence, with post-translation modifications indicated. Residues indicated in black were previously identified, and residues indicated in red indicate phosphorylation sites we have identified here via mass spectrometry of immunoprecipitated GLIS3 long and short isoforms from a mouse β-cell line, using βTC-6 cells. (**B**) Luciferase assay of GLIS3 and indicated phosphomutants in βTC-6 cells using a mouse insulin promoter (MIP)-driven luciferase. Error bars indicate SEM, N = 3. (**C**) Schematic of our RIME experiments using βTC-6 cells.

We have previously reported that GLIS3 undergoes several post-translational modifications (PTMs), including phosphorylation, methylation, and ubiquitination [28], while another study reported GLIS3 sumoylation [29]. PTMs can be cell type- and condition-dependent, adding to the complexity of studying them. An additional mass spectrometry analysis of GLIS3 (both the long and short proteins) in the mouse pancreatic β-cell line βTC-6 confirmed several of these PTMs and identified at least 13 additional phosphorylation sites (Figure 5A, red aa). Interestingly, several PTM sites localize within the 140 to 210 aa region. This includes S165, S179, S196, and R193, which, according to publicly available data, are phosphorylated or methylated under some conditions in mice and humans. The serine 154 residue is followed by a proline and may therefore also be a potential candidate for phosphorylation, while K176 could be a potential methylation, acetylation, ubiquitination, or sumoylation site. However, mutations of S154, S165, S179 (either individually or simultaneously), S196, K176, and R193 to alanine did not affect the transactivation activity of the long isoform (Supplementary Figure S3).

To determine whether several downstream phosphorylation sites identified through mass spectrometry had a role in regulating GLIS3 activity, the effect of individual point mutations to alanine on GLIS3 transcriptional activity was analyzed with the GLISBS-Luc reporter assay. None of the single mutations had a significant effect on GLIS3 transactivation potential (Figure 5B). It is possible that the phosphorylation of two or more amino acids acts in concert to control GLIS3 activity or function in a promoter context-dependent manner, as has been reported for other transcription factors [30]. Alternatively, under the conditions tested, some of these sites may not be phosphorylated, and consequently a mutation to alanine would not have an effect.

In addition to PTMs, RIME (Figure 5C) identified several new GLIS3-interacting proteins (Supplementary Tables S2 and S3) in addition to the few reported previously [31,32,33,34]. This included proteins that are known to be part of co-activator (e.g., EP300, TRRAP, NCOA1, MED16) or co-repressor (e.g., SIN3A, NCOR1, NACC1, HDAC3) complexes. This is consistent with our previous studies showing that GLIS3 can activate and repress gene transcription [1,2]. Consistent with previous studies showing that GLIS3 can be ubiquitinated and sumoylated, RIME further identified several proteins (e.g., USP7, PIAS2, CUL2, USP34, TRIM33) involved in ubiquitination/sumoylation, suggesting possible mechanisms for regulating the protein stability of both GLIS3 isoforms. The different isoforms, post-translational modifications, and protein interactions together contribute to the complexity of the regulatory mechanisms that control GLIS3 activity and physiological functions, as well as its role in disease.

## 4. Discussion

Great insights have been obtained about the biological functions of GLIS3 and its regulatory role in various pathologies, including diabetes, hypothyroidism, polycystic kidney disease, and cancer [7,9]. Transcriptome and cistrome analyses not only identified the genes regulated by GLIS3 but also revealed the causal mechanisms for these diseases. However, little is known about the mechanisms that regulate GLIS3 expression and activity. In this study, we identified several processes that together likely govern GLIS3 expression and/or activity, and consequently its regulation of physiological processes and its role in disease. We show that, in all mouse tissues tested, the *Glis3* gene undergoes AS and generates two transcripts that produce two different isoforms, a longer 935 aa GLIS3 protein and a shorter, more abundant 659 aa isoform. While we have focused our analysis here on the mouse isoforms of *Glis3*, other organisms are predicted to have similar isoforms. An analysis of the publicly available RNA-seq data from pigs, rhesus monkeys, dogs, and rabbits confirmed the presence of both exon 2-3 and exon 2-4 AS events in at least one tissue (Supplementary Figure S4). AS is a fundamental post-transcriptional regulatory mechanism that adds to the complexity of gene regulation. Most multi-exon genes undergo some form of AS. Transcripts generated by AS have been reported to differ in their stability and rate of translation, and can function as noncoding RNAs, while respective protein isoforms can differ in their localization, protein stability, and protein–protein interactions [17].

In this study, we show that the shorter GLIS3 isoform exhibits a greater transactivation ability that is at least in part due to an increased protein stability (Figure 3). The human *GLIS3* gene can also generate two transcripts, but only in the testis, while other tissues express only the long transcript. This is of particular interest, as our lab previously identified a role for GLIS3 in germ cell development in the testis [35]. Indeed, germ cell development relies on a coordinated process involving multiple mechanisms, including alternative splicing [36]. Whether the shorter and longer isoforms have distinct functions in germ cell development remains to be established. Moreover, future studies are needed to identify a specific protein involved in the alternative splicing of *GLIS3*. While the long human isoform is analogous to the mouse long isoform, the short human isoform is distinct from the mouse isoform and is 155 amino acids shorter than the human long isoform. In contrast to mouse GLIS3, the two human isoforms did not exhibit a difference in transactivation activity when analyzed in HEK293T cells, suggesting that these 122 aa are not involved in regulating GLIS3 stability. However, there are several caveats: GLIS3 stability or transactivation may be, respectively, cell type- or promoter context-dependent, and translation in humans could start at a more downstream methionine and produce a more stable and active protein.

While their stability differed, both mouse isoforms appear to localize to the nucleus, as the nuclear localization signal for GLIS3 is located within the DNA binding domain common to both isoforms. This suggests that the differences in isoform activity may be due in part to protein stability. Our data indicated that the increased stability of the short mouse GLIS3 isoform appears to be related to a removal of the 276 aa N-terminus in the long GLIS3 isoform. Through deletion analysis, this region could be narrowed down to the sequence between aa 140 and 210 of the long GLIS3 isoform (Figure 4A). Since the human short form, which starts at methionine 155, does not differ in protein stability from the long isoform, the region could be confined to aa 155-210. This region of GLIS3 is not well conserved with other members of the GLI/GLIS family, indicating that the function of this region may be unique to GLIS3.

Degrons, regions that regulate protein stability, are often controlled by PTMs, including acetylation, methylation, and/or phosphorylation [37]. We previously reported that GLIS3 protein is ubiquitinated and sumoylated; however, the ubiquitination and sumoylation sites were outside the 155-210 region [29]. Mass spectrometry showed that GLIS3 is greatly phosphorylated containing at least 33 phosphorylated aa and several methylated aa (Figure 5A) [28]. Several of these PTMs were localized within the 155-210 region. However, individual point mutations of these PTMs did not affect the transactivation ability of GLIS3 (Supplementary Figure S3). It is possible that the phosphorylation of two or more aa acts in concert to control GLIS3 activity or function in a promoter context-dependent manner as has been reported for other transcription factors [30,38]. Additionally, we have yet to identify the appropriate kinases/phosphatases responsible for the regulation of these PTMs, which are likely cell type-specific, further adding to the complexity of GLIS3 regulation. Alternatively, the loss of the 276 aa N-terminus may alter the conformation of GLIS3, which affects the interaction of ubiquitin ligases with downstream degrons (e.g., VKQE^226^ [29]). Adding further to the complexity is the likelihood of the presence in cells of multiple GLIS3 proteoforms that differ in their abundance and combination of PTMs and which may have distinct effect on GLIS3 activity and function.

Dysregulated splicing events have been implicated in various pathologies, including cancer and neurological disorders [25,26]. AS also plays a critical role in the regulation of pancreatic β cell functions, and its dysregulation has been associated with the pathophysiology of pancreatic cells and shown to increase during islet inflammation [27]. GLIS3 is critical for the generation and regulation of pancreatic β cell functions, while deficiency in GLIS3 causes neonatal diabetes [4,5,6,9,10,11,19]. This raised the possibility that changes in GLIS3 isoform expression may have a role in the pathophysiology of diabetes. However, we did not observe a difference in the relative expression of *Glis3* isoforms between wild-type and *db*/*db* mice, a model for Type 2 diabetes (Supplementary Figure S2). It is possible that *Glis3* alternative splicing is regulated under different developmental or pathological conditions. Considering the multitude of tissues and developmental stages in which *Glis3* is expressed, future studies across all tissues might provide insights into the regulation and functions of the two isoforms.

In addition to PTMs, RIME analysis identified several proteins that are part of a GLIS3 protein complex. This included various transcription factors, co-activators, co-repressors, methyltransferases, ubiquitin ligases, and several proteins of unknown function (Supplementary Tables S2 and S3). We previously reported that GLIS3 can act as an activator as well as a repressor of target gene transcription [8,9,19]. Some of the proteins identified in our MS analysis have been found to interact and form complexes, such as SIN3A, SIN3B, NCOR2, MORF4L1, HDAC3, and HDAC1, which form the SMRT or NCOR complexes [39]. This complex has roles both as a co-activator and co-repressor, depending on its genomic context [40]. We have yet to identify any differences in the interaction between these co-regulators and the previously mentioned GLIS3 isoforms, although such differences may exist in cell type- and context-specific manners. As we learn more about the function of these complexes in various tissues and disease states, more light will be shed on the mechanism by which GLIS3 receives upstream signaling and regulates its target genes.

In summary, our study identifies AS as an additional mechanism for regulating *Glis3* gene expression and activity. In addition, mass spectrometry identified many new PTMs and a series of additional GLIS3-associated proteins, including co-repressors or co-activators involved in methylation, ubiquitination, or sumoylation. We hypothesize that these processes together control GLIS3 expression, activity, and its regulation of physiological processes, as well as its role in disease.

## Data Availability

Mass Spectrometry data have been deposited in MassIVE data repository, Accession number: MSV000099318. RNA-seq data from previous studies are available from the Gene Expression Omnibus with the following accession #s: GSE122120, GSE207775, and GSE240532.

## Supplementary Materials

The following supporting information can be downloaded at https://www.mdpi.com/article/10.3390/cells14231912/s1. Supplementary Figure S1. Gel electrophoresis of qRT-PCR results indicates production of only one band. Supplementary Figure S2. No change in the relative *Glis3* isoform expression in *db*/*db* mice. Supplementary Figure S3. Mutations of individual aa within GLIS3 140-210 do not affect activity. Supplementary Figure S4. Species comparison of *Glis3* alternative splicing. Supplementary Table S1. qPCR and Cloning primers. Supplementary Table S2. Proteins identified from Flag-tagged and HA-tagged GLIS3 immunoprecipitation using a local version of the Spectrum Mill MS Proteomics Software (Rev BI.08.01.217). Supplementary Table S3. Proteins identified from HA-tagged GLIS3 immunoprecipitation using a Mascot (version 2.2.07) search within the Thermo Proteome Discoverer software (version 2.5.0.400).

## Abbreviations

The following abbreviations are used in this manuscript:

AS	Alternative Splicing
GLISBS	GLIS Binding Site
