# Supplementary material for: Alternative Splicing (AS) Provides an Alternative Mechanism for Regulating GLIS3 Expression and Activity
Source: Cells. 2025 Dec 2;14(23):1912. doi: 10.3390/cells14231912 (PMC12691133; doi:10.3390/cells14231912)
Supplement: Supplementary file 1 [file cells-14-01912-s001.zip › Supplementary Figures.pptx]

## Slide 1
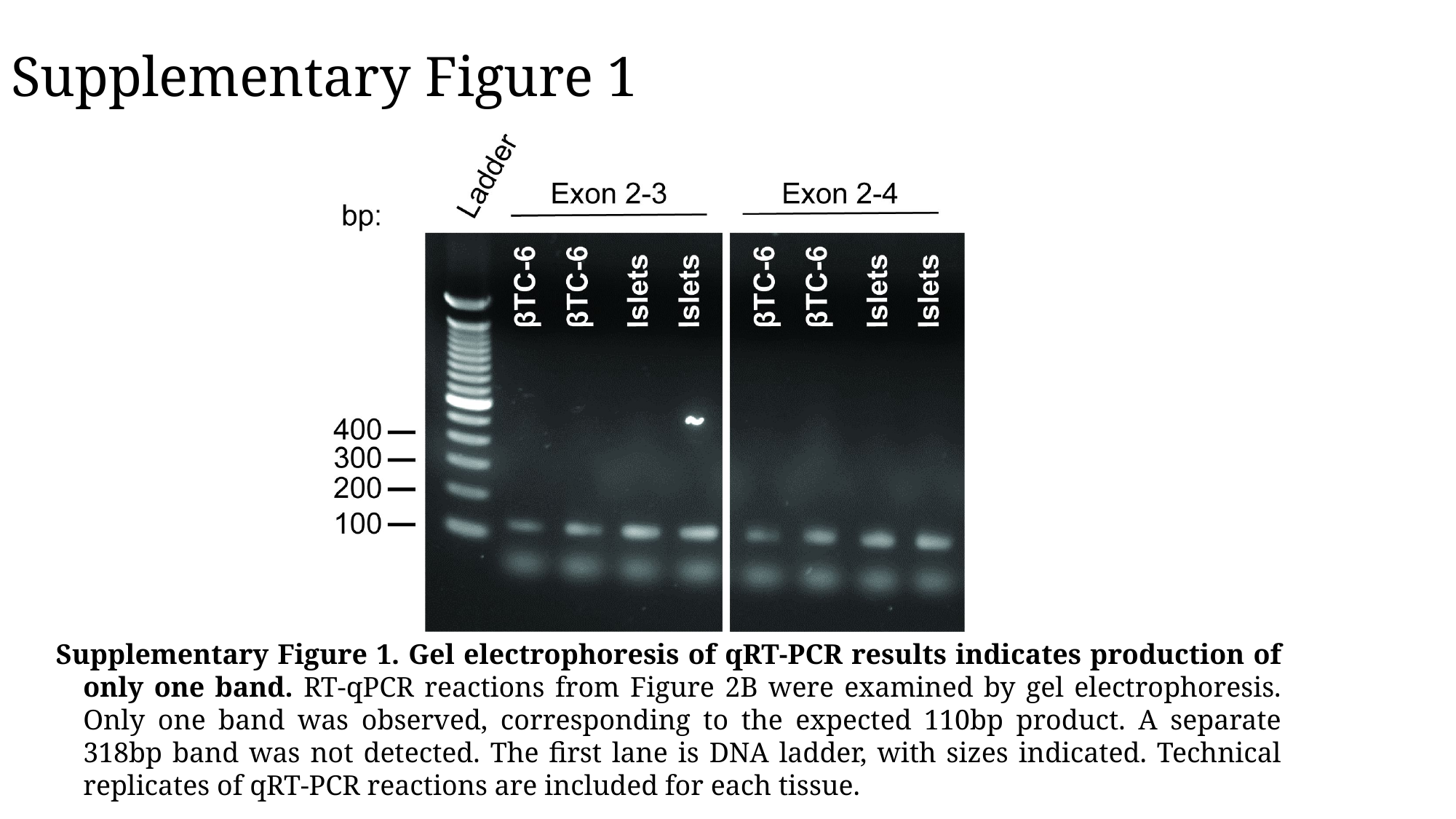

# Supplementary Figure 1
Supplementary Figure 1. Gel electrophoresis of qRT-PCR results indicates production of only one band. RT-qPCR reactions from Figure 2B were examined by gel electrophoresis. Only one band was observed, corresponding to the expected 110bp product. A separate 318bp band was not detected. The first lane is DNA ladder, with sizes indicated. Technical replicates of qRT-PCR reactions are included for each tissue.

## Slide 2
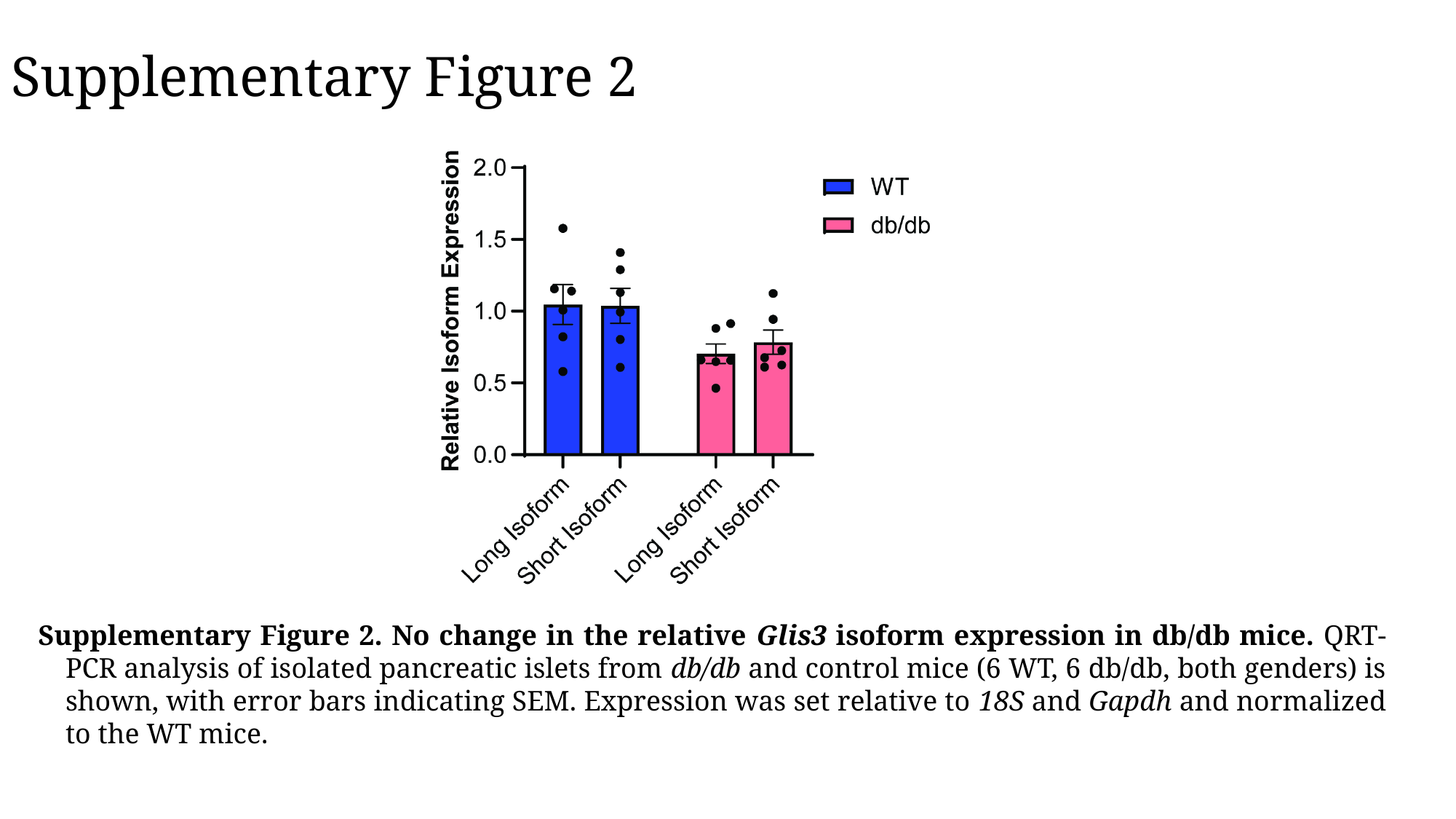

Supplementary Figure 2
Supplementary Figure 2. No change in the relative Glis3 isoform expression in db/db mice. QRT-PCR analysis of isolated pancreatic islets from db/db and control mice (6 WT, 6 db/db, both genders) is shown, with error bars indicating SEM. Expression was set relative to 18S and Gapdh and normalized to the WT mice.

## Slide 3
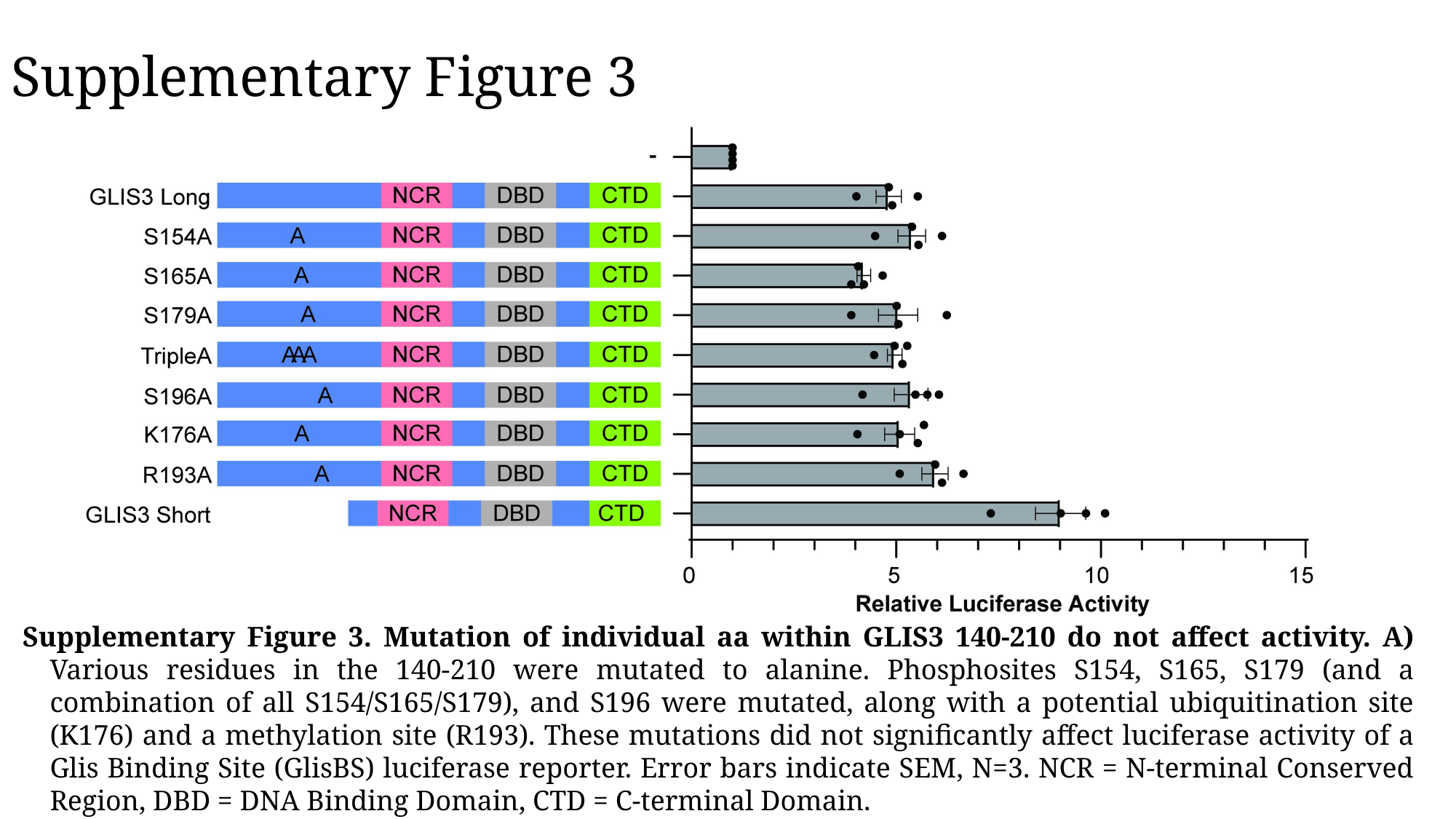

Supplementary Figure 3
Supplementary Figure 3. Mutation of individual aa within GLIS3 140-210 do not affect activity. A) Various residues in the 140-210 were mutated to alanine. Phosphosites S154, S165, S179 (and a combination of all S154/S165/S179), and S196 were mutated, along with a potential ubiquitination site (K176) and a methylation site (R193). These mutations did not significantly affect luciferase activity of a Glis Binding Site (GlisBS) luciferase reporter. Error bars indicate SEM, N=3. NCR = N-terminal Conserved Region, DBD = DNA Binding Domain, CTD = C-terminal Domain.

## Slide 4
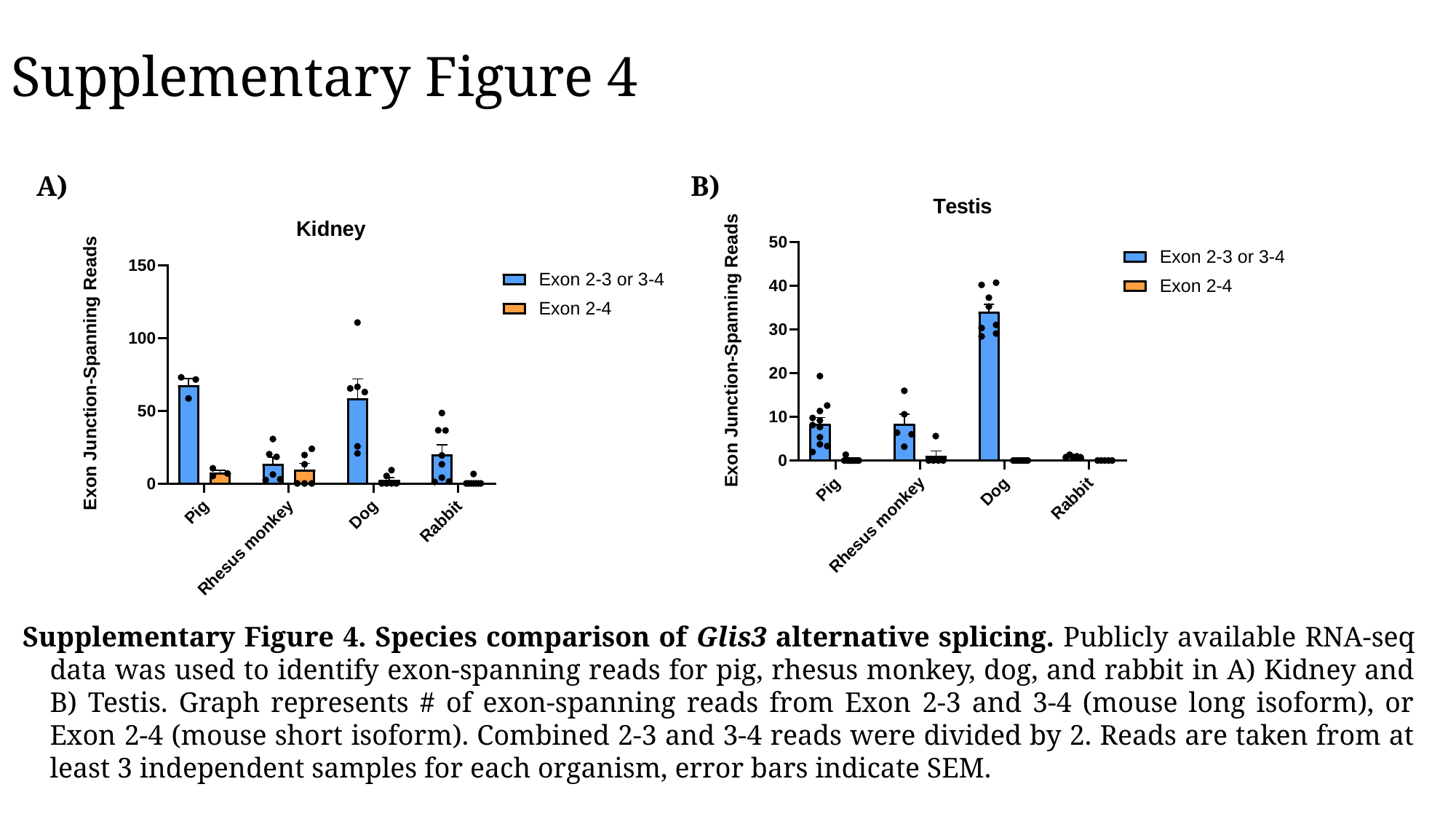

# Supplementary Figure 4
A)
B)
Supplementary Figure 4. Species comparison of Glis3 alternative splicing. Publicly available RNA-seq data was used to identify exon-spanning reads for pig, rhesus monkey, dog, and rabbit in A) Kidney and B) Testis. Graph represents # of exon-spanning reads from Exon 2-3 and 3-4 (mouse long isoform), or Exon 2-4 (mouse short isoform). Combined 2-3 and 3-4 reads were divided by 2. Reads are taken from at least 3 independent samples for each organism, error bars indicate SEM.
